# Integration of intratumoral/peritumoral radiomics and deep learning for predicting overall survival in non-small cell lung cancer patients: a multicenter study

**DOI:** 10.3389/fonc.2025.1669200

**Published:** 2025-11-20

**Authors:** Yongxin Liu, Yuteng Pan, Qiusheng Wang, Huayong Jiang, Na Lu, Diandian Chen, Yanjun Yu, Yanxiang Gao, Huijuan Zhang, Yinglun Sun, Jianfeng Qiu, Fuli Zhang

**Affiliations:** 1 Radiation Oncology Department, The Seventh Medical Center of Chinese People's Liberation Army PLA General Hospital, Beijing, China; 2 School of Radiology, Shandong First Medical University and Shandong Academy of Medical Sciences, Taian, China; 3 School of Automation Science and Electrical Engineering, Beihang University, Beijing, China; 4 The First Affiliated Hospital of Shandong First Medical University, Jinan, China

**Keywords:** non-small cell lung cancer, CT, radiomics, deep learning, overall survival

## Abstract

**Background:**

Prognostic assessment of non-small cell lung cancer (NSCLC) relies on TNM staging, yet tumor heterogeneity limits its accuracy. This study aimed to develop a model for improving the prediction of overall survival (OS) in NSCLC patients receiving radiotherapy, which integrated intratumoral/peritumoral radiomics features and 3D deep learning (DL) features.

**Methods:**

A total of 303 NSCLC patients from three centers were retrospectively enrolled. Radiomics features were extracted from intratumoral and 3/6/9 mm peritumoral regions on CT scans. A network named 3D-SE-ResNet was proposed to extract DL features. Lasso-Cox and principal component analysis (PCA) were used to integrate multidimensional features to establish a combined model. Performance was evaluated via the concordance index (C-index) and area under the curve (AUC). Survival differences were visualized through Kaplan–Meier curves.

**Results:**

The 6 mm expansion peritumoral radiomics features analysis showed the best performance (C-index: 0.63). The DL features outperformed the radiomics features (C-index: 0.74 vs 0.63). The combined model achieved the highest accuracy (C-index: 0.77/0.73 across datasets). K–M analysis confirmed significant survival differences (log-rank P < 0.001).

**Conclusion:**

The combined model integrates intratumoral/peritumoral radiomics features and 3D DL features and effectively predicts the OS of NSCLC patients, offering a novel tool for personalized radiotherapy strategies.

## Introduction

Non-small cell lung cancer (NSCLC) is a leading cause of cancer-related mortality worldwide, accounting for 80%–85% of all lung cancer cases (1–3). Radiotherapy (RT) plays a pivotal role in the therapeutic management of NSCLC patients. However, the current 5-year survival rate remains below 30% (4–6), highlighting the urgent need for precise prognostic stratification to optimize RT strategies and adjuvant therapeutic decisions.

The conventional TNM staging system has limited predictive accuracy because of its inability to capture the heterogeneity of the tumor microenvironment (7, 8). Radiological assessment is limited in quantifying tumor information because of its heavy reliance on subjective experience. Radiomics has shown substantial potential in tumor prognosis prediction (9–11). Accumulated evidence indicates that the peritumoral lung parenchyma, a key pathway for tumor dissemination, is closely linked to recurrence and poor prognosis (12–14). Peritumoral radiomics, which quantifies heterogeneity in surrounding regions, has been shown to effectively capture microenvironmental dynamics and enhance prognostic predictions (15–17). Nevertheless, the optimal expansion distance of peritumoral regions of interest (ROIs) remains controversial across studies, which may hinder their clinical generalizability (18–20).

The integration of artificial intelligence (AI) with radiomics offers a novel direction for prognostic modeling. Compared with conventional 2D convolutional neural networks (CNNs), 3D CNNs can more comprehensively capture three-dimensional spatial heterogeneity and have demonstrated superiority in tasks such as pulmonary nodule classification (21–23). However, limitations exist, including overfitting risks due to limited medical imaging datasets and a shortage of networks optimized for survival analysis. We propose a novel network architecture named 3D-SE-ResNet, which embeds a squeeze-and-excitation (SE) module into the 3D ResNet framework to achieve adaptive feature channel calibration. Furthermore, this study establishes a novel paradigm for personalized prognosis by synergistically integrating intratumoral/peritumoral radiomics features with 3D deep learning (DL) features through a principal component analysis (PCA)-driven fusion strategy.

This study aimed to construct a multicenter-validated combined model for predicting overall survival (OS) in NSCLC patients undergoing RT by integrating intratumoral/peritumoral radiomics features and 3D DL features. These advancements aim to provide a robust tool for optimizing individualized radiotherapy strategies.

## Materials and methods

### Patients data

This multicenter retrospective study included 303 non-small cell lung cancer (NSCLC) patients from three centers. The training set included 203 adenocarcinoma and squamous cell carcinoma patients from the publicly available MAASTRO Clinic dataset (24), with the study dates (from the metadata file) ranging from November 2004 to January 2014. The test set included 100 histologically confirmed adenocarcinoma or squamous cell carcinoma patients enrolled between January 2016 and August 2021 (68 from the Seventh Medical Center of Chinese PLA General Hospital and 32 from Shandong Provincial Hospital Affiliated with Shandong First Medical University), all of whom met the following criteria.

The inclusion criteria were as follows: (1) histologically verified NSCLC (adenocarcinoma or squamous cell carcinoma); (2) aged >18 years; (3) received radiotherapy or chemoradiotherapy; (4) available radiotherapy planning CT scans; and (5) complete clinical records. The exclusion criteria included the following: (1) the presence of other primary malignancies; (2) poorly defined tumor boundaries; and (3) loss to follow-up.

**Figure 1:**
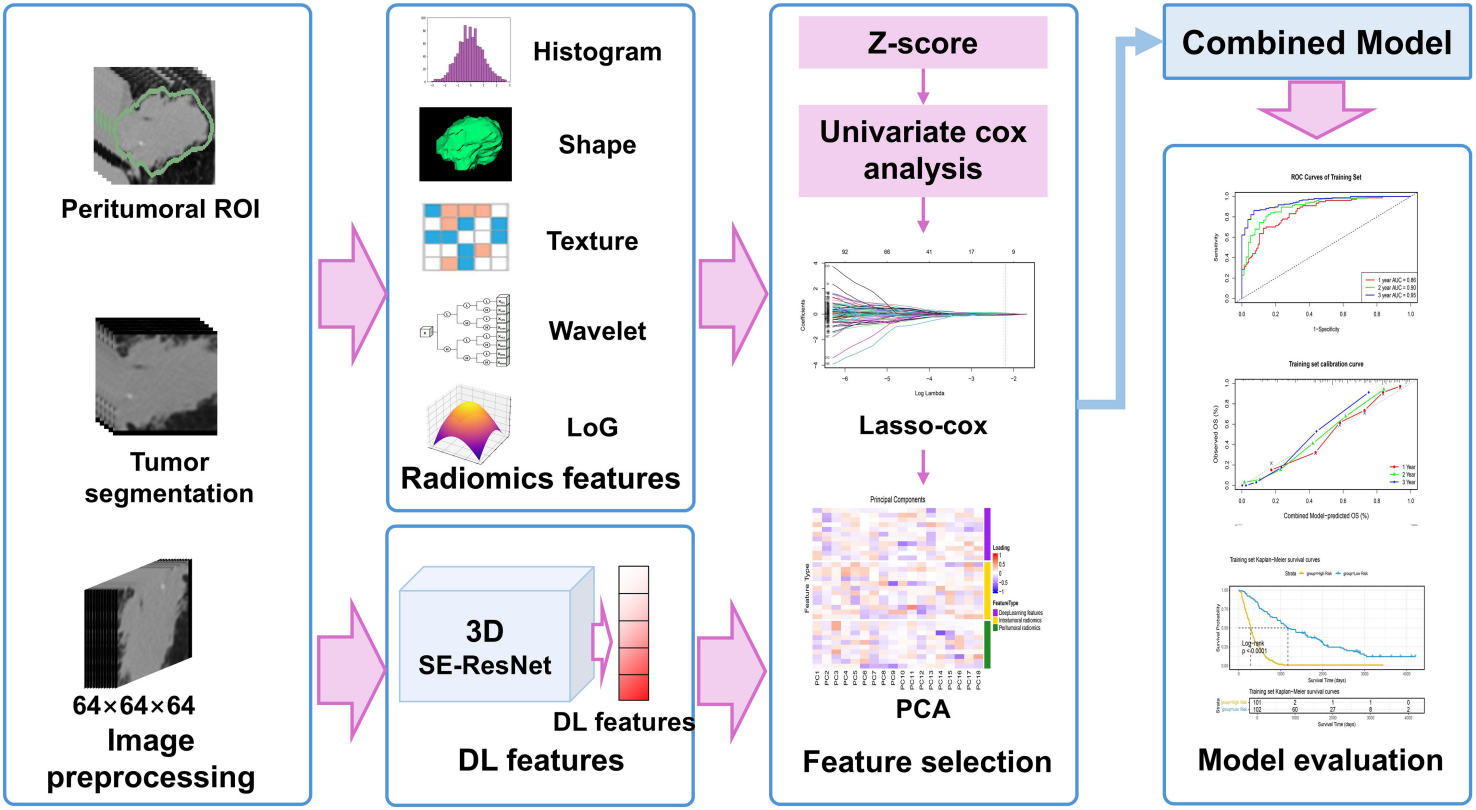
Study design and workflow: DL, deep learning; LoG, Laplacian of Gaussian; PCA, principal component analysis; ROI, regions of interest; SE, Squeeze-and-Excitation.

The protocol was conducted in compliance with the Declaration of Helsinki and has been approved by the Ethics Committee of the Seventh Medical Center of Chinese PLA General Hospital (No. S2025-030-01) and the Ethics Committee of Shandong First Medical University (No. SB-KJCX2101). Owing to the retrospective nature of the study and the use of anonymized data, written informed consent was waived. The study workflow is shown in **Figure 1**.

### Treatment and follow-up

In the training set, patients with stage N2/N3 and T4 disease were required to first receive 3 cycles of induction chemotherapy with gemcitabine combined with cisplatin or carboplatin, and radiotherapy was initiated at least 14 days after the completion of chemotherapy. All radiotherapy was performed in accordance with the principles that mean lung dose (MLD)≤ 19 Gy and the maximum spinal cord dose ≤ 54 Gy, with a maximum radiation dose of 79.2 Gy. Among these patients, a fractionation schedule of 1.8 Gy per fraction (twice daily) was adopted for those undergoing radical radiotherapy with a minimum of 8 h between the two fractions. Three dimensional conformal radiotherapy (3D-CRT) or intensity modulated radiotherapy (IMRT) techniques were used. For patients undergoing concurrent chemoradiotherapy, 2 cycles of chemotherapy were completed first, followed by irradiation of 45 Gy at 1.5 Gy per fraction (twice daily); thereafter, the fractionation schedule was adjusted to 2.0 Gy per fraction (once daily) for a boost irradiation of 6–24 Gy.

All patients in the test set underwent definitive radiotherapy (RT) or chemoradiotherapy. The RT target volume covered the primary lesion and involved lymph nodes, with a conventional fractionation schedule: 2 Gy per fraction, five fractions per week, and a total dose of 50–60 Gy. RT techniques were implemented according to the clinical routines of the participating centers: the Seventh Medical Center of Chinese PLA General Hospital adopted intensity-modulated radiotherapy (IMRT) or volumetric modulated arc therapy (VMAT), while Shandong Provincial Hospital used IMRT. For VMAT, two full arcs were used; for IMRT, 5–7 coplanar fields were used. For patients undergoing chemoradiotherapy, platinum-based dual-drug combination regimens were mainly adopted: etoposide combined with cisplatin (EP regimen) was preferred for patients with squamous cell carcinoma; pemetrexed combined with cisplatin or carboplatin (PC regimen) was recommended for patients with non-squamous cell carcinoma; in addition, paclitaxel combined with carboplatin was also an alternative regimen for cisplatin-intolerant patients.

The baseline clinical characteristics included sex, age, histologic subtype, TNM stage, and overall stage. The study’s major outcome measure was OS, which was calculated from the commencement of RT to either mortality occurrence or censoring at the final follow-up (August 2024). Patients in the test set were followed every 3 months during the initial postdiagnosis year and every 6–12 months thereafter, with a minimum follow-up of 36 months. Survival outcomes for the training set were derived from the publicly available dataset (24).

### Image acquisition and preprocessing

RT planning CT scans were performed on Philips and Siemens large-bore simulation scanners, covering the thoracic region with a tube voltage of 120 kV, slice thickness of 2–5 mm, and a reconstruction matrix of 512×512. To mitigate variability from multicenter imaging protocols, CT images were preprocessed as follows: DICOM files were converted to NIFTI format, followed by isotropic resampling with B-spline interpolation to standardize the voxel dimensions to 1×1×1 mm^3^.

### ROI segmentation and radiomics feature extraction

The intratumoral region of interest (ROI) was defined as the gross tumor volume (GTV) by an experienced radiation oncologist (working for over 20 years). To evaluate the prognostic impact of peritumoral regions, three concentric peritumoral expansions (3/6/9 mm) were generated via SimpleITK (v 2.3.1). Radiomics features were extracted using PyRadiomics (v 3.1) with the following parameters: intensity normalization enabled and a bin width of 25 for histogram discretization. A total of 1316 radiomics features were extracted from each ROI, encompassing five categories: first-order statistics (n=18), shape-based features (n=14), texture features (n=75), wavelet-filtered features (n=744), and Laplacian of Gaussian (LoG)-filtered features (n=465, with sigma values set to [1.0, 2.0, 3.0, 4.0, 5.0]).

### Deep learning network establishment

**Figure 2:**
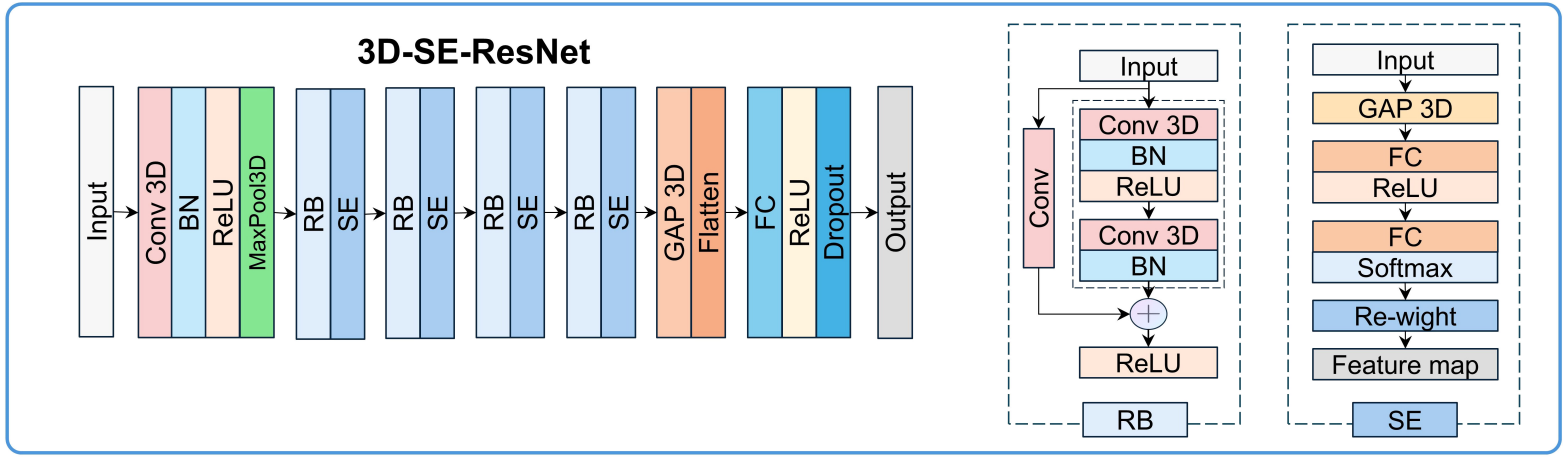
3D-SE-ResNet framework diagram: BN, batch normalization; Conv, convolution layer; FC, fully connected layer; GAP, global average pooling layer; RB, residual block.

The 3D-SE-ResNet architecture was systematically adapted from ResNet18 to accommodate 3D survival analysis tasks. As illustrated in **Figure 2**, the network comprises three core components: (1) a backbone module containing an initial 3D convolutional layer (kernel size=3×3×3, stride=2) for spatial downsampling and preliminary feature extraction, which is followed by a 3D max-pooling layer (kernel size = 3×3×3, stride = 2) to further reduce spatial dimensions. (2) Four sequential residual blocks with a progressively increasing number of filters (64, 128, 256, 512). Each residual block contains two 3D convolutional basic units, both utilizing 3×3×3 kernels combined with batch normalization and ReLU activation, and employs skip connections to mitigate the gradient vanishing problem. The first unit’s convolutional layer in the first residual block acts directly on the input, while the second unit’s convolutional layer follows a “BN+ReLU+convolution” architecture; both units use a convolution stride of 1. In subsequent residual blocks, the first unit’s convolution uses a stride of 2, and the second unit uses a stride of 1, with both units adopting the “BN+ReLU+convolution” architecture for their convolutional layers. (3) A squeeze-and-excitation (SE) module integrated after each residual block to implement the channel attention mechanisms.

The SE module performs feature recalibration through three steps (with a compression ratio of 64): (1) Spatial compression: Generating channel-wise statistics via global average pooling (GAP), transforming each 3D feature map (H×W×D×C, where C is the number of channels) into a 1D vector (1×1×1×C). (2) Channel dependency modeling: Establishing inter-channel dependencies through two fully connected layers. The first fully connected layer reduces the dimension to C/64 with ReLU activation, and the second restores the dimension to C with sigmoid activation, generating channel weights (range: 0–1). (3) Feature map rescaling: Adaptive enhancement of prognosis-relevant channels and suppression of irrelevant signals via element-wise multiplication between the original feature maps and the learned channel weights. The network terminates with fully connected layers (512→512→256→128 units), dropout (rate=0.4), and L2 regularization (λ=1e-4). The output layer uses tanh activation for survival prediction. Ablation experiments were conducted to validate the contribution of the SE module.

### Deep learning network training and feature extraction

The model training encompassed three phases: preprocessing, augmentation, and optimization. Intratumoral ROIs were resized to 64×64×64 via linear interpolation and normalized via Z score transformation. To increase data diversity, seven types of 3D augmentations were implemented, including contrast adjustment, brightness scaling, gamma correction, Gaussian noise addition, blurring, 3D mirroring, and spatial rotation, with the technical specifications provided in **Table 1**. Network optimization employs the Cox negative partial likelihood loss function (25), which is optimized via the Adam optimizer with an initial learning rate of 1×10^-4^, exponentially decayed by a factor of 0.96 every 1000 steps, and a batch size of 32. Training proceeded for 100 epochs with early stopping triggered if the validation loss plateaued for 15 consecutive epochs. The feature vectors (512-dimensional) were extracted from the GAP layer post training. All the implementations utilized TensorFlow 2.7 on an NVIDIA GeForce RTX 4070 Ti GPU with CUDA 11.2 acceleration.

**Table 1:** Technical details of 3D data augmentation.

Augmentation type	Parameter settings	Probability
Contrast Adjustment	Multiplier range (1.0-1.75)	15%
Brightness Multiplicative Adjustment	Scaling factor (0.7-1.5)	15%
Gamma Correction	γ range (0.5-2.0)	15%
Gaussian Noise	Variance range (0-0.05)	15%
Gaussian Blur	σ range (0.5-1.5)	15%
3D Mirroring	Random flipping along X/Y/Z axes	30%
3D Spatial Rotation	Rotation (0-360°) around Z-axis	30%

### Feature selection and model development

The feature selection procedures were restricted to the training set. For radiomics and deep learning features, a three-step process was applied: (1) Z score normalization to standardize feature scales; (2) univariate cox analysis (*P* < 0.05) to identify survival-associated features; and (3) least absolute shrinkage and selection operator (Lasso) Cox regression with standard 10-fold cross-validation to retain nonzero coefficients. The feature selection workflow is illustrated in **Figure 1**. A radiomics/deep learning signature was derived via a linear combination of selected features and their coefficients.

Principal component analysis (PCA) was performed on the integrated intratumoral/peritumoral radiomics and deep learning features, retaining components accounting for ≥ 95% of the cumulative variance. Factor loading heatmaps were used to visualize the contribution of each feature to the principal components. Within the training set, the dimensionality-reduced features were integrated to construct the combined predictive model.

### Model validation and evaluation

Model discrimination was assessed via Harrell’s concordance index (C-index) and area under the curve (AUC) from receiver operating characteristic curve (ROC) analysis. Calibration curves (bootstrap resampling: 1000 iterations) were used to evaluate the agreement between the predicted and observed survival probabilities. Patients were stratified into low/high-risk subgroups through the median risk score in the training set to determine the optimal cutoff value.

To further evaluate the model’s utility in personalized risk stratification, subgroup analyses were conducted. Specifically, we stratified patients by overall stage (Stage I - II vs. Stage III), histologic subtype (adenocarcinoma vs. squamous cell carcinoma), gender (male vs. female), and age (≥ 65 years vs. < 65 years) in both the training and test sets. For each subgroup, survival differences between cohorts were analyzed via the Kaplan–Meier method with log - rank tests, and the corresponding Kaplan–Meier curves were plotted.

### Statistical analysis

Statistical computations were conducted with R v4.4.0 and Python 3.9. Categorical variables were evaluated via Fisher’s exact test or the chi-square test; for continuous variables, normality was first determined using the Shapiro-Wilk test, with the independent t test applied for normally distributed variables and the Mann-Whitney U test for non-normally distributed variables. DeLong’s test was used to compare differences in AUC values between the single signature and combined models. Subgroup analyses stratified by clinical characteristics (tumor stage, histological type, age, and gender) were performed in both the training and testing sets; for survival-related outcomes, the log-rank test was employed to compare survival differences between subgroups. All hypothesis tests were two-tailed with a significance threshold set at α=0.05.

## Results

### Patient cohort characteristics

A total of 303 non-small cell lung cancer (NSCLC) patients from three centers were included in this study. The baseline clinical characteristics of all patients are listed in **Table 2**.

**Table 2:** Clinical characteristics of all patients.

Characteristics	Training set (n=203)	Test set (n=100)	*P* value
Age	69 (63–77)	66 (57–72)	0.01
Sex			0.09
Male	144(70.9%)	80(80.0%)	
Female	59(29.1%)	20(20.0%)	
Histology			0.03
Adenocarcinoma	51(25.1%)	42(42.0%)	
Squamous	152(74.9%)	58(58.0%)	
T Stage			0.30
1	37(18.2%)	20(20.0%)	
2	77(37.9%)	32(32.0%)	
3	32(15.8%)	24(24.0%)	
4	57(28.1%)	24(24.0%)	
N Stage			0.12
0	81(39.9%)	26(26.0%)	
1	16(7.9%)	10(10.0%)	
2	65(32.0%)	37(37.0%)	
3	39(19.2%)	27(27.0%)	
4	2(1.0%)	0(0%)	
M Stage			0.10
0	202(99.5%)	98(98.0%)	
1	0(0.0%)	2(2.0%)	
3	1(0.5%)	0(0%)	
Overall Stage			0.22
I	34(16.7%)	14(14.0%)	
II	31(15.3%)	13(13.0%)	
IIIA	58(28.6%)	28(28.0%)	
IIIB	80(39.4%)	45(45.0%)	
Survival Time (days)	493 (252–1217)	786 (509–1024)	0.03

### Radiomics feature analysis

**Figure 3:**
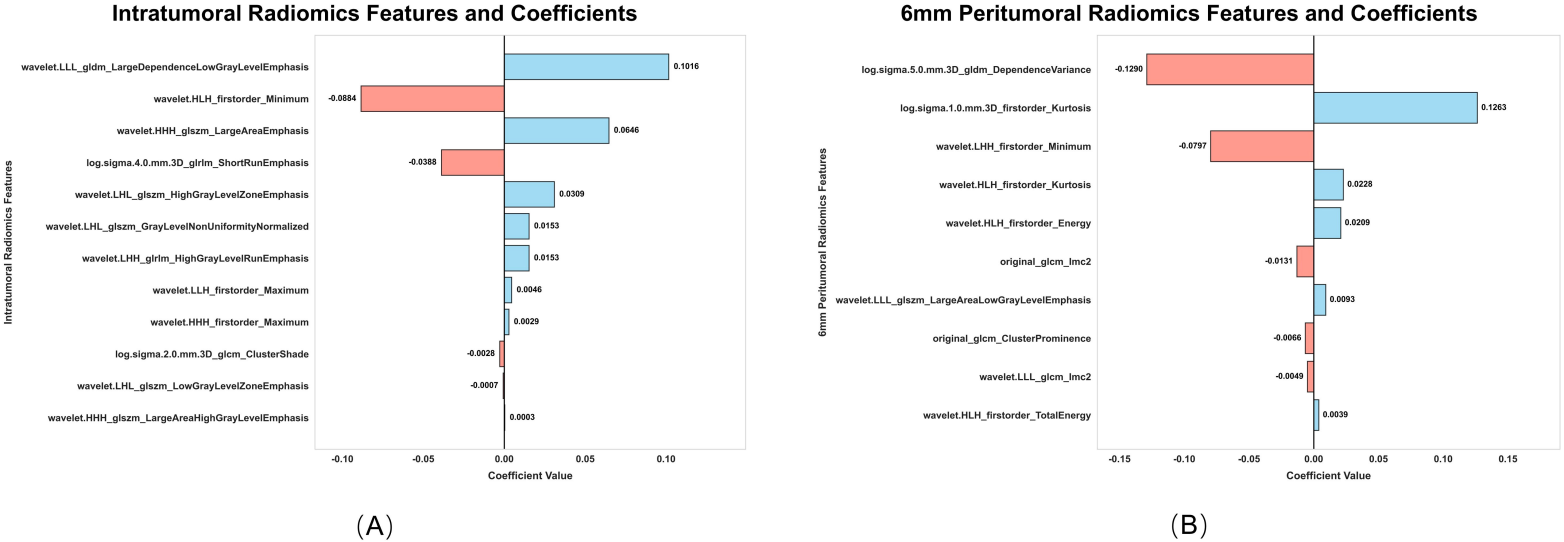
Intratumoral **(A)** and 6-mm peritumoral **(B)** radiomics features and coefficients.

From the intratumoral and peritumoral regions (3 mm, 6 mm, and 9 mm expansions), 1316 radiomics features were extracted. Univariate cox regression identified 214 significant intratumoral radiomics features and 83, 88, and 125 peritumoral radiomics features, respectively. Subsequent Lasso-Cox analysis further refined these features to 12 intratumoral and 9, 10, and 10 peritumoral key radiomics features. To explore the synergistic effects of the intratumoral and peritumoral regions, combined radiomics signatures were generated by integrating selected radiomics features from both regions. Predictive performance varied significantly across peritumoral expansion distances and their combinations (**Table 3**). Among the standalone peritumoral radiomics signatures, the 6-mm expansion radiomics signature achieved the highest C-index (0.63, 95% CI: 0.58–0.67), whereas the combined Rad+Rad_6 mm radiomics signature further improved the C-index (0.65, 95% CI: 0.60–0.69); thus, this radiomics signature was selected as the standardized peritumoral region. The names and corresponding coefficients of all features within the Rad + Rad_6 mm radiomics signature are presented in **Figure 3**.

**Table 3:** Comparison of C-index (95%CI) for intratumoral/peritumoral radiomics signatures with different expansion distances.

Signatures	Training set	Test set
Rad	0.63 (0.58-0.68)	0.61 (0.48-0.68)
Rad_3mm	0.61 (0.57-0.65)	0.61 (0.49-0.68)
Rad_6mm	0.63 (0.58-0.67)	0.62 (0.49-0.69)
Rad_9mm	0.61 (0.57-0.65)	0.59 (0.46-0.66)
Rad+Rad_3mm	0.64 (0.60-0.69)	0.63 (0.52-0.70)
Rad+Rad_6mm	0.65 (0.60-0.69)	0.64 (0.53-0.70)
Rad+Rad_9mm	0.64 (0.59-0.69)	0.62 (0.51-0.71)

Rad: Intratumoral radiomics signature. Rad_3mm, Rad_6mm, Rad_9mm: Peritumoral radiomics signatures with 3 mm, 6 mm, and 9 mm expansion distances, respectively.

### Deep learning feature analysis

**Figure 4:**
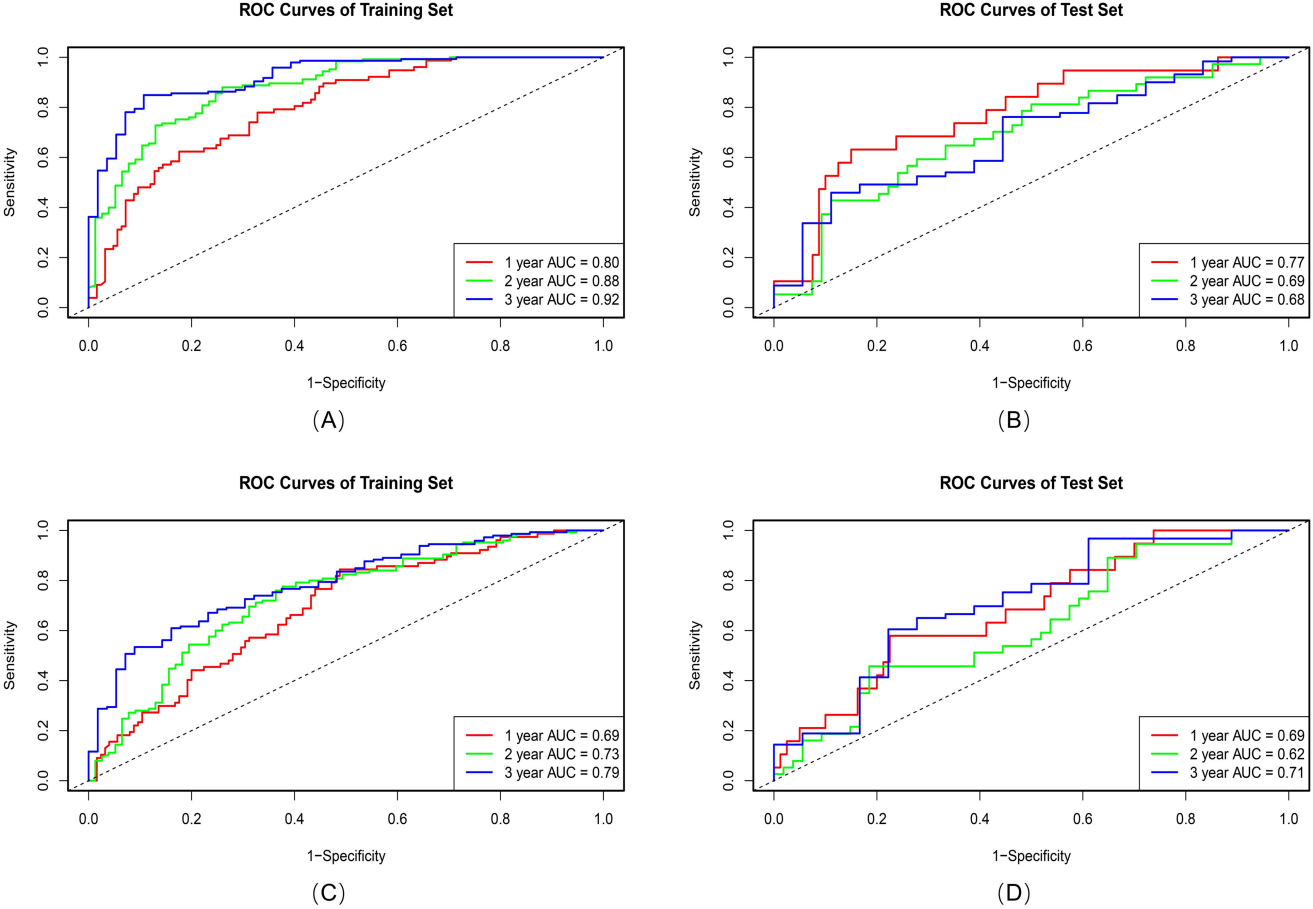
ROC curves of the 3D-SE-ResNet **(A, B)** and 3D ResNet **(C, D)** DL signatures on training and test sets.

Among 512 deep learning (DL) features extracted from 3D-SE-ResNet, 11 key prognostic features were retained after univariate cox and Lasso-Cox screening. Compared with the intratumoral and 6-mm peritumoral radiomics signatures, the DL signature showed a trend of improved discriminative performance, with a C-index of 0.74 (95% CI: 0.70-0.77) in the training set and 0.66 (95% CI: 0.58–0.73) in the test set. Ablation experiments confirmed the critical role of the SE module: its integration yielded a 13.8% performance gain in the 3D-ResNet model (C-index: 0.74 vs. 0.65), indicating that the SE module effectively enhances the extraction of prognosis-related features. As shown in **Figure 4**, the AUC values of the 3D-SE-ResNet DL signature for predicting 1-, 2-, and 3-year overall survival were higher than those of the baseline model at all timepoints.

### Combined model development and validation

**Figure 5:**
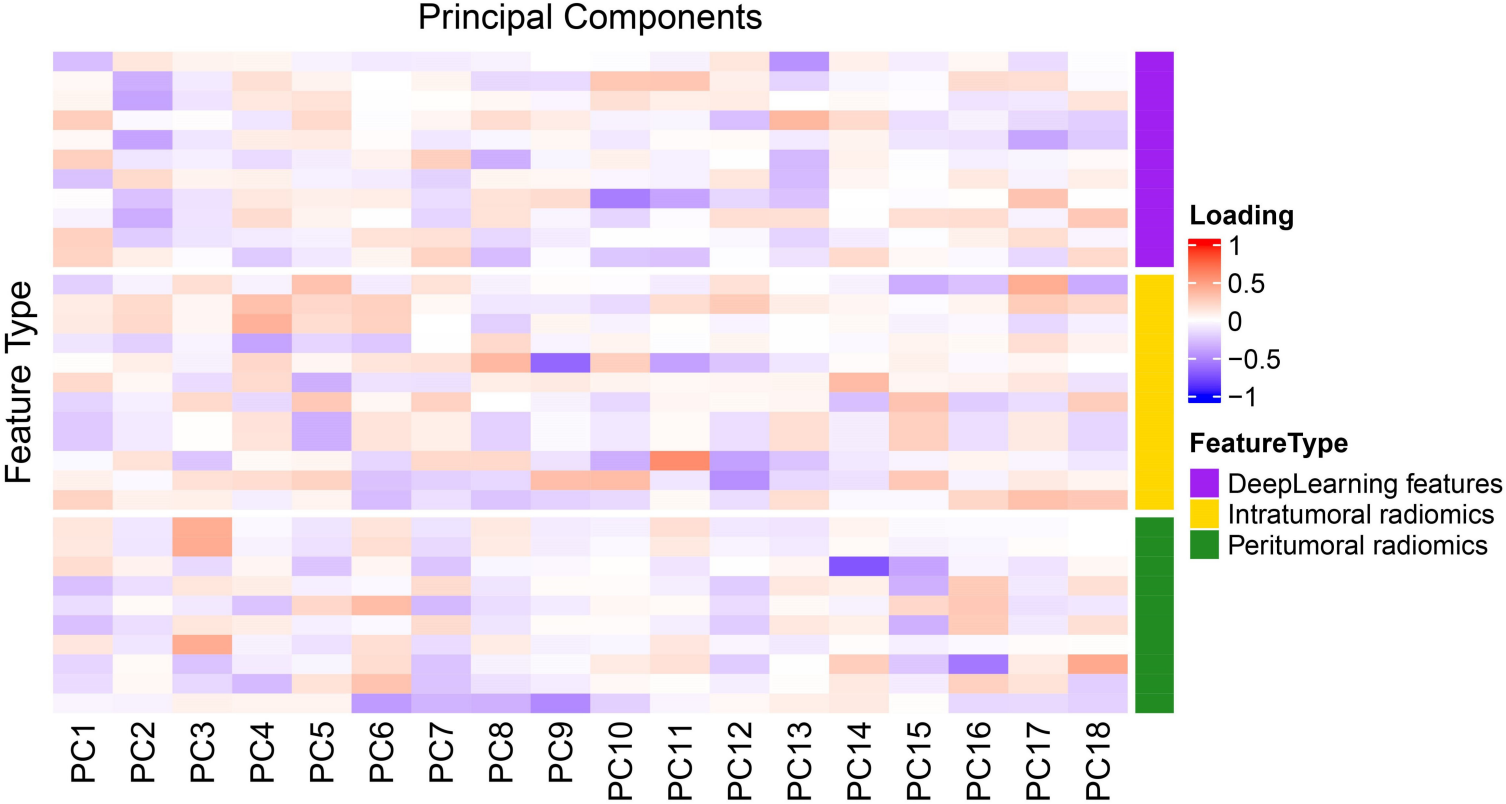
PCA factor loading heatmap.

**Figure 6:**
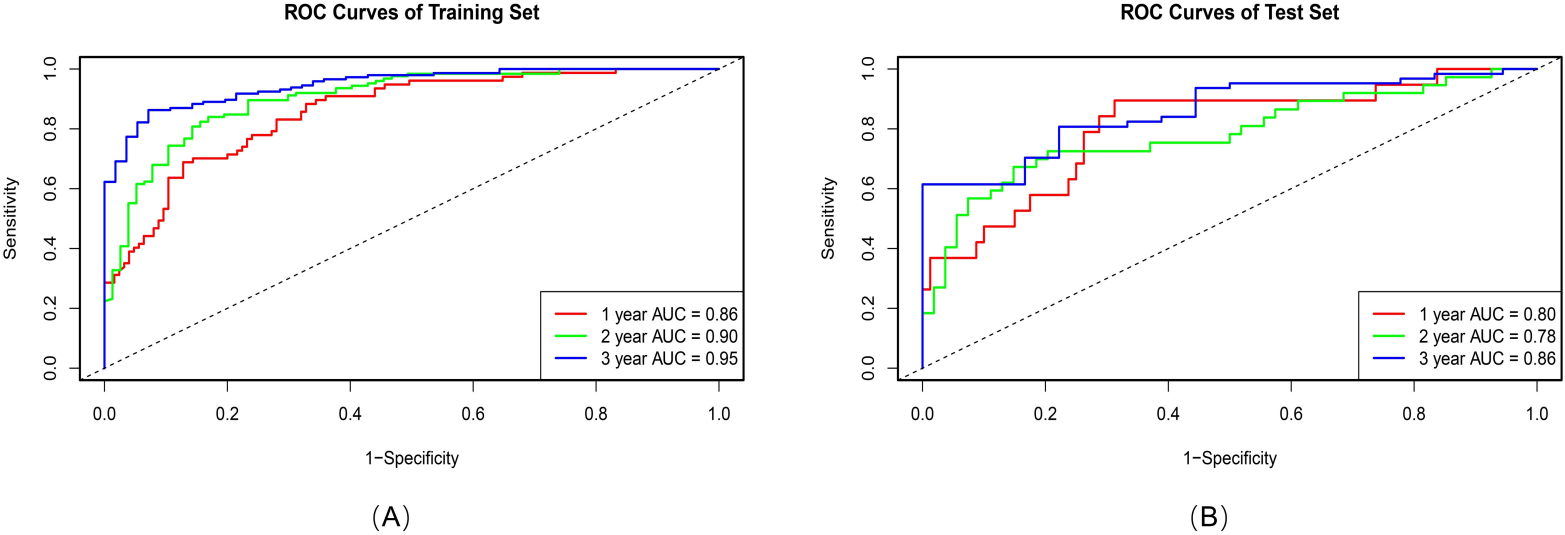
ROC curves of the Combined model in the training set **(A)**, and test set **(B)**.

Principal component analysis (PCA) was applied to integrate intratumoral radiomics, 6-mm peritumoral radiomics, and DL features, with the distribution of feature contributions across principal components visualized in **Figure 5**. The combined model, which integrates PCA-reduced features, achieved C-index values of 0.77 (95% CI: 0.75–0.81) in the training set and 0.73 (0.72–0.86) in the test set, outperforming individual signatures. ROC analysis revealed AUCs of 0.86 (95% CI: 0.81–0.90), 0.90 (0.86–0.94), and 0.95 (0.92–0.97) for predicting 1-, 2-, and 3-year OS, respectively (**Table 4**), with the corresponding ROC curves illustrated in **Figure 6**.

**Table 4:** AUC (95%CI) of intratumoral/peritumoral radiomics signatures, deep learning signatures, and combined model for 1-, 2-, and 3-year OS in patients.

Models	AUC	Training set	Test set	*P* value
Rad	1-year	0.68 (0.61-0.75)	0.64 (0.50-0.78)	0.03
	2-year	0.69 (0.61-0.76)	0.64 (0.52-0.75)	0.02
	3-year	0.76 (0.68-0.83)	0.66 (0.50-0.81)	<0.01
Rad_6mm	1-year	0.70 (0.63-0.77)	0.64 (0.50-0.79)	0.01
	2-year	0.71 (0.63-0.77)	0.71 (0.60-0.82)	0.2
	3-year	0.71 (0.63-0.79)	0.67 (0.53-0.80)	0.02
DL	1-year	0.80 (0.74-0.85)	0.77 (0.66-0.88)	0.5
	2-year	0.88 (0.83-0.92)	0.69 (0.58-0.80)	0.05
	3-year	0.92 (0.88-0.96)	0.68 (0.55-0.82)	< 0.01
Combined Model	1-year	**0.86** **(0.81-0.90)**	**0.80** **(0.69-0.91)**	**—**
	2-year	**0.90** **(0.86-0.94)**	**0.78** **(0.68-0.88)**	**—**
	3-year	**0.95** **(0.92-0.97)**	**0.86** **(0.77-0.94)**	**—**

Boldface values indicate the best predictive performance.

The *P* values from the DeLong test compare individual signatures with the Combined Model in the test set.

### Risk stratification and survival analysis

**Figure 7:**
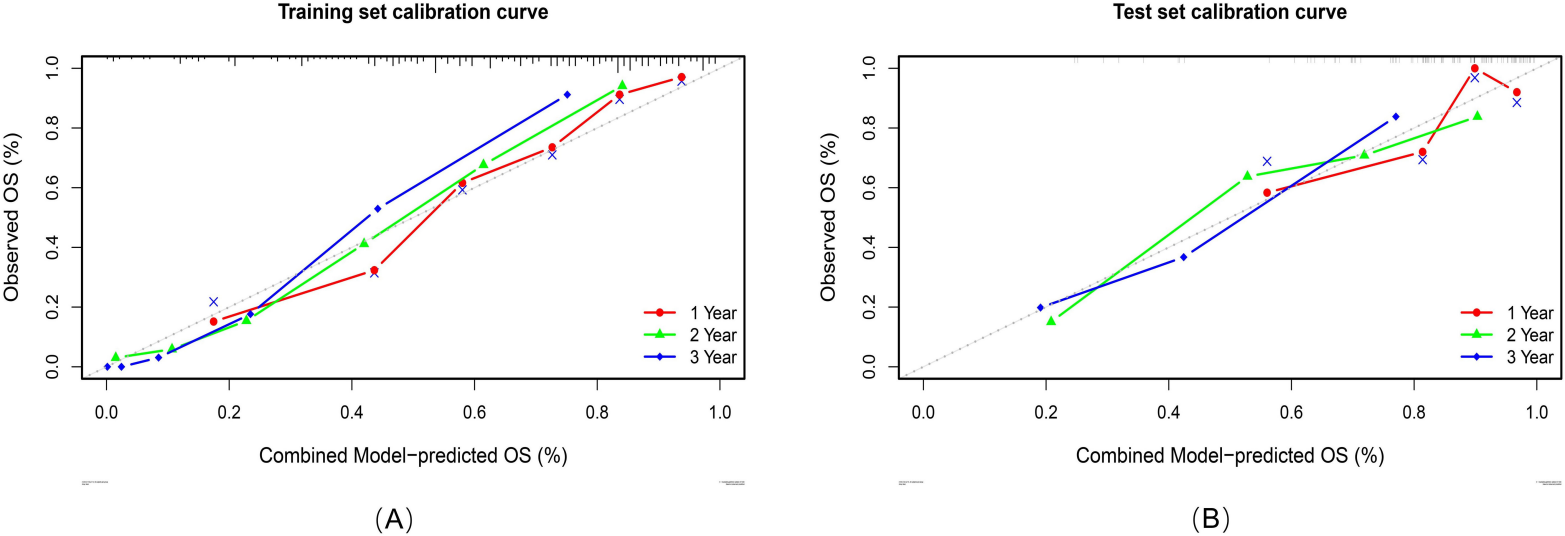
Calibration curves of the combined model in the training set **(A)** and test set **(B)**.

**Figure 8:**
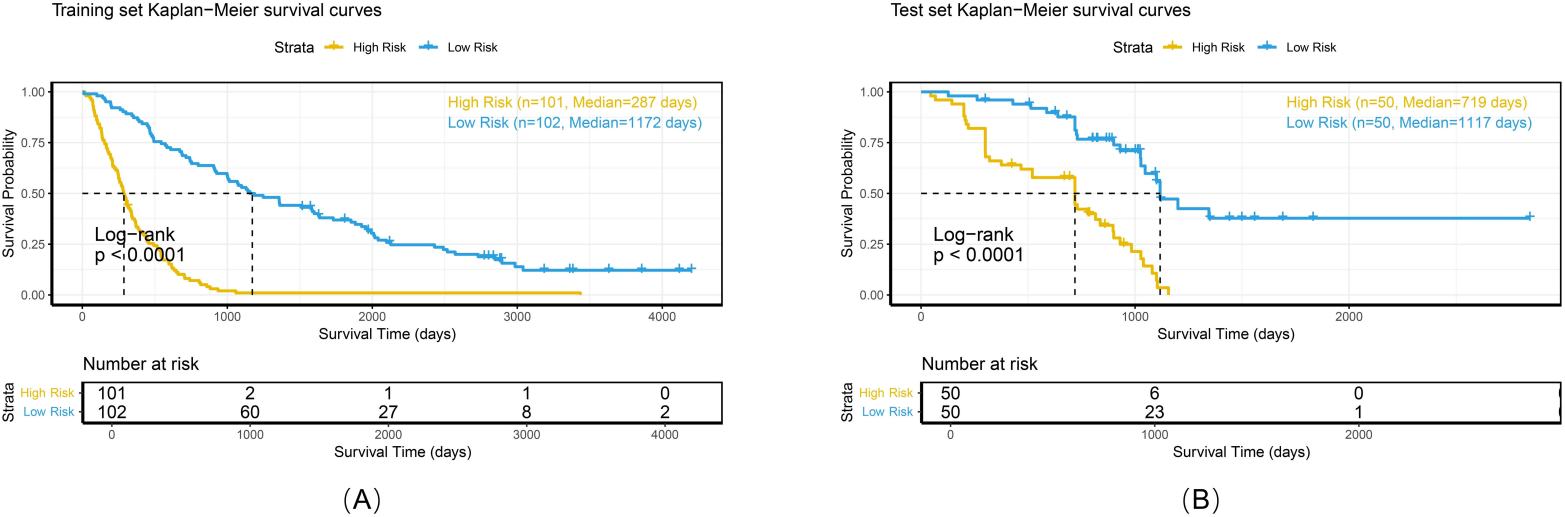
Kaplan-Meier survival curves of the combined model in the training set **(A)**, and test set **(B)**.

Calibration analyses revealed high concordance between the predicted and observed survival probabilities (**Figure 7**). Patients were stratified into high-risk and low-risk groups by applying a risk threshold (cutoff value = 1.14), which was derived from the combined model’s median risk score. The high-risk group had a significantly poorer prognosis compared to the low-risk group in both datasets: the hazard ratio (HR) for the training set was 5.39 (95% CI: 3.83–7.59), and for the test set was 4.48 (95% CI: 2.54–7.91). Kaplan–Meier analysis further confirmed significant survival differences between the two risk groups (log-rank P < 0.0001 for both datasets; **Figure 8**).

**Figure 9:**
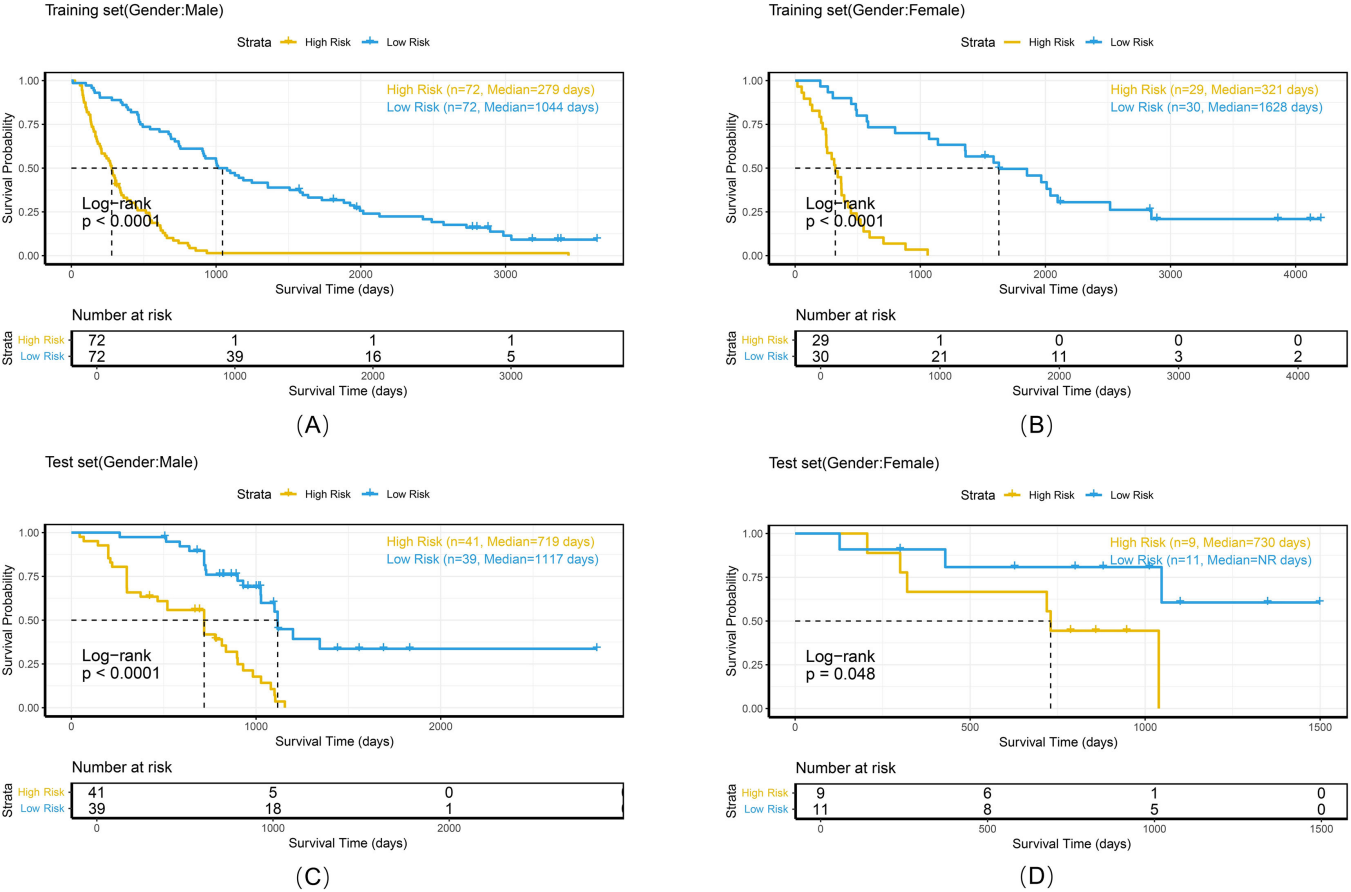
Kaplan-Meier survival curves of the combined model stratified by gender in the training set **(A, B)** and test set **(C, D)**.

**Figure 10:**
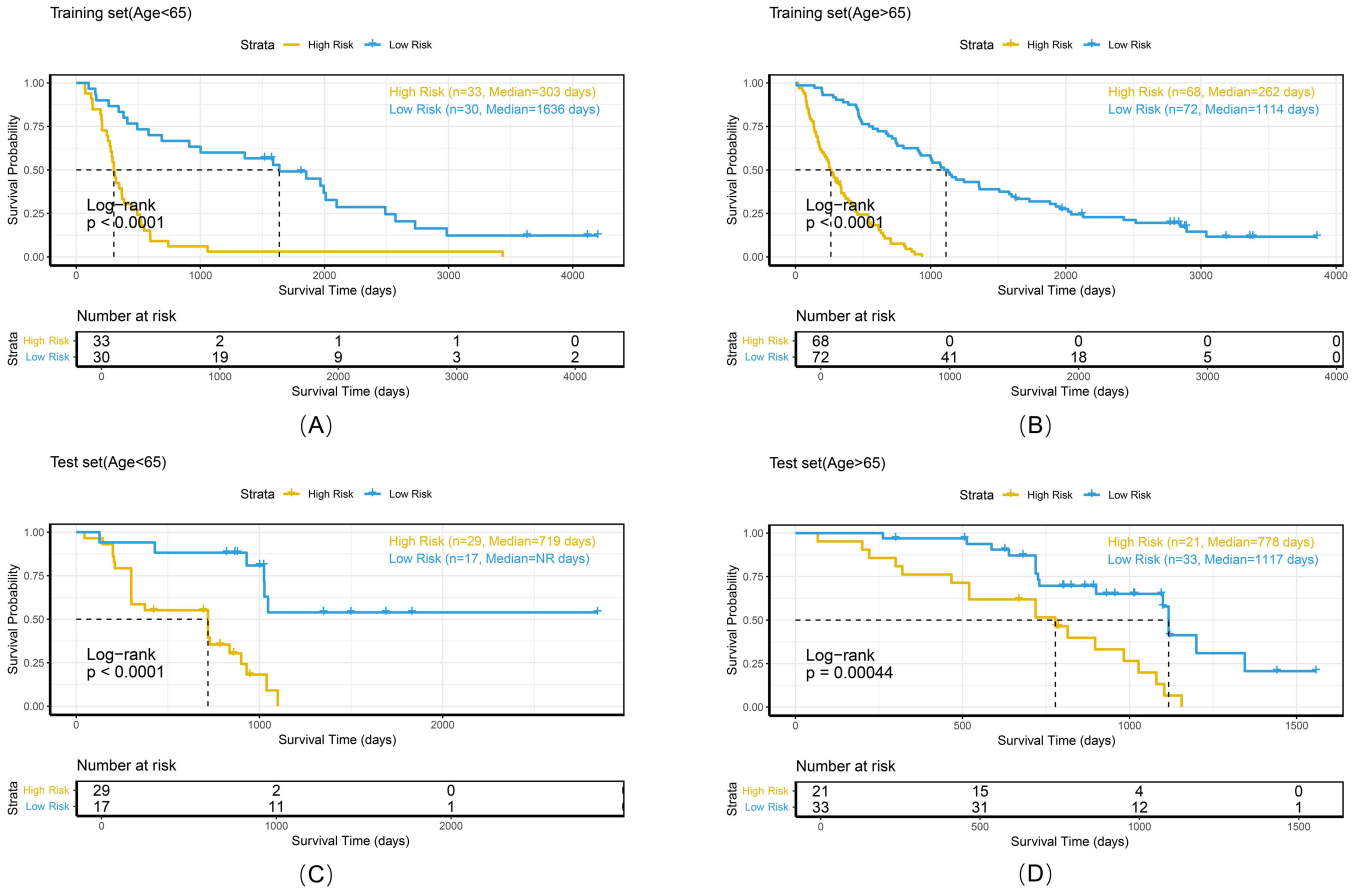
Kaplan-Meier survival curves of the combined model stratified by age in the training set **(A, B)** and test set **(C, D)**.

**Figure 11:**
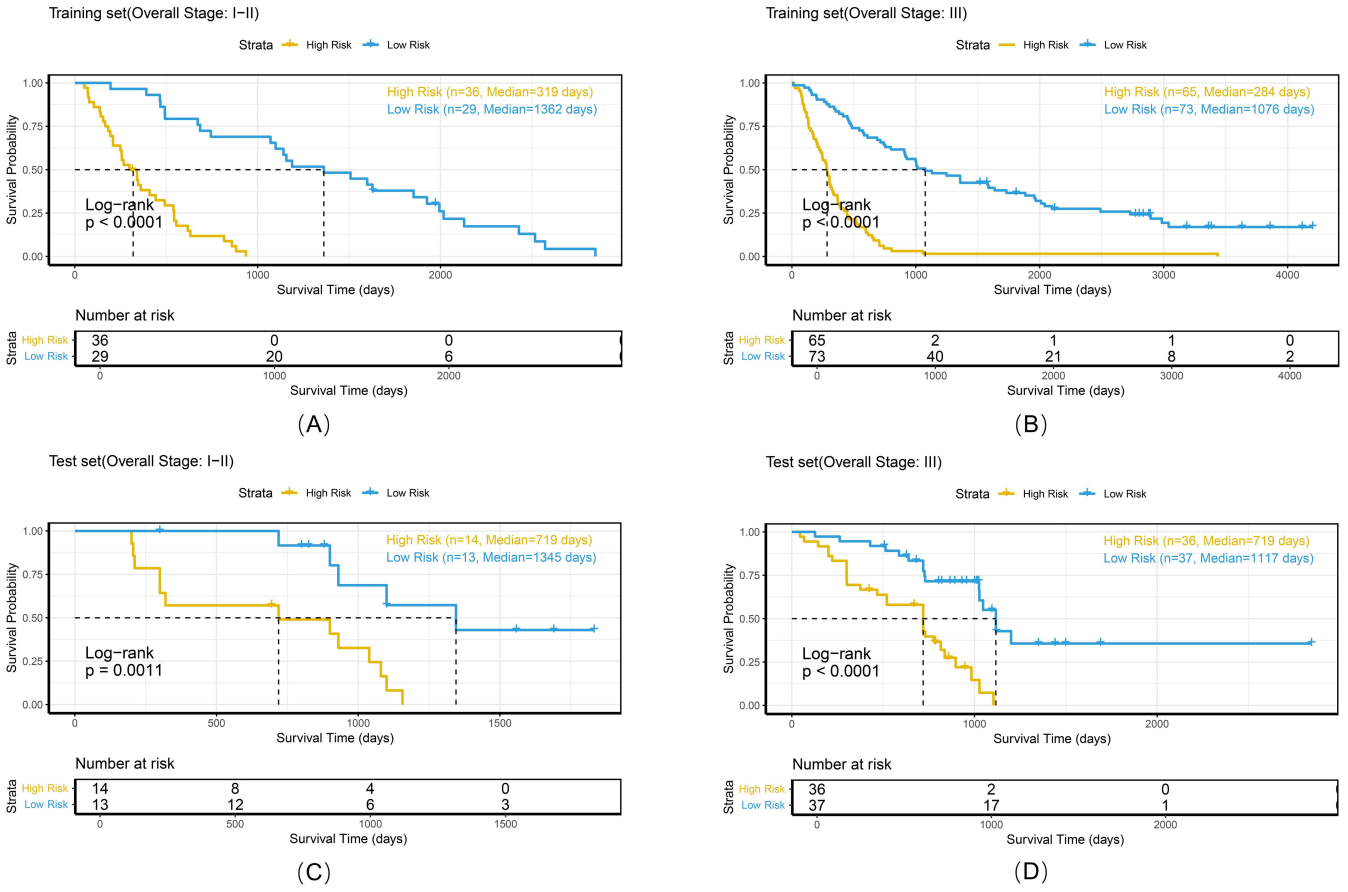
Kaplan-Meier survival curves of the combined model stratified by histologic subtype in the training set **(A, B)** and test set **(C, D)**.

**Figure 12:**
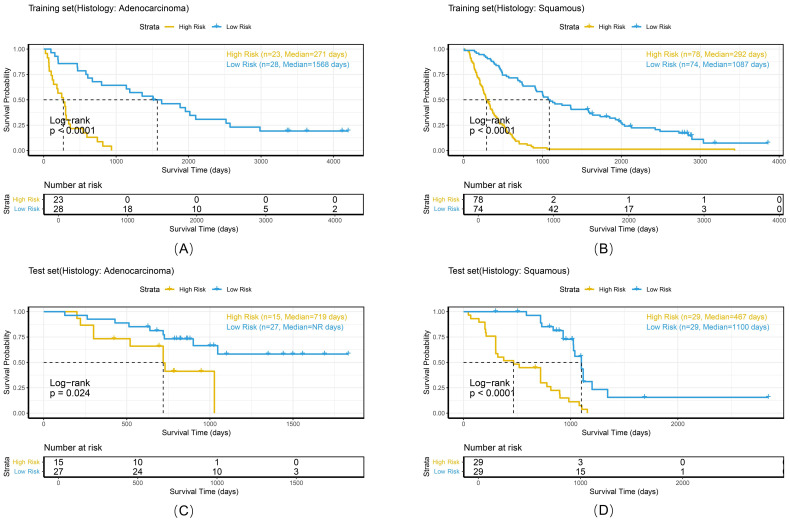
Kaplan-Meier survival curves of the combined model stratified by overall stage in the training set **(A, B)** and test set **(C, D)**.

To comprehensively evaluate the personalized risk stratification ability of the combined model, subgroup analyses were performed within clinically relevant strata. The model demonstrated consistent risk discrimination capabilities across gender subgroups (**Figure 9**), age subgroups (**Figure 10**), histologic subtype subgroups (**Figure 11**), and overall stage subgroups (**Figure 12**). Statistically significant survival differences were observed between high - and low - risk groups in both the training and test sets (log - rank test, P < 0.05), validating its robustness across patient populations with diverse clinical characteristics.

## Discussion

This study developed a multicenter-validated combined model that integrates intratumoral/peritumoral radiomics features and 3D deep learning features to predict overall survival (OS) in non-small cell lung cancer (NSCLC) patients undergoing radiotherapy. The results demonstrated that radiomics features from the 6-mm peritumoral region exhibited optimal prognostic value (C-index=0.63), whereas deep learning (DL) features extracted by 3D-SE-ResNet significantly outperformed conventional radiomics features (C-index=0.74 vs. 0.63). The combined model achieved a C-index of 0.73 in the test set and an AUC of 0.86 for 3-year OS prediction, outperforming the conventional TNM staging system (AUC≈0.65) (26, 27) and validating the efficacy of the multidimensional feature fusion strategy. These findings provide a novel framework for individualized prognostic assessment in NSCLC radiotherapy.

The aggressive nature of NSCLC leads to destruction of adjacent lung structures, leading to the infiltration of vasculo-lymphatic cancer at tumor margins (28). This complex tumor–host interface is often overlooked in traditional intratumoral radiomics (29). Liu et al. (30) demonstrated that peritumoral regions within 3–9 mm from the tumor boundary contain biological information relevant to lung cancer heterogeneity, suggesting that bidirectional tumor–host interactions at the interface critically influence prognosis. Through systematic evaluation of peritumoral regions at varying extents (3/6/9 mm), we identified the 6-mm peritumoral zone as optimal for balancing microenvironmental information capture and noise reduction (C-index=0.63), with further performance improvement when this zone was combined with intratumoral features (C-index=0.65). These findings indicate that peritumoral regions provide complementary information to intratumoral areas, supporting the hypothesis that peritumoral regions serve as pathways for tumor invasion (12, 15). Compared with single-center studies (31), our multicenter validation confirms the generalizability of peritumoral radiomics, suggesting its clinical applicability.

3D CNNs enable precise spatial characterization of tumors through the integration of continuous cross-sectional information (32, 33). Wang et al. demonstrated the superior performance of 3D DL features over 2D methods in predicting occult lymph node metastasis in patients with laryngeal squamous cell carcinoma (AUC = 0.89 vs 0.86) (21). However, tumor heterogeneity leads to significant variations in prognostic contributions from different feature channels, and conventional CNNs lack the ability to prioritize critical features. To address this limitation, we innovatively integrated the SE module into the 3D ResNet framework. The SE module compresses spatial dimensions via global average pooling and learns channelwise weights through fully connected layers. This process achieves adaptive recalibration of feature maps, enhancing the expression of prognosis-relevant information while suppressing redundant signals (34). Ablation experiments demonstrated the critical role of the SE module: the 3D-SE-ResNet model exhibited a 13.8% improvement in the C-index (0.74 vs. 0.65) compared to the baseline 3D-ResNet, highlighting its ability to resolve spatial heterogeneity through attention-driven feature calibration.

The hierarchical representation capability of deep neural networks enables 3D-SE-ResNet to capture complex tumor–host interface patterns that are challenging to quantify with traditional handcrafted features, corroborating prior studies highlighting deep learning’s advantages in high-dimensional feature representation (35, 36). The combined model achieved increased predictive accuracy by synergistically integrating intratumoral/peritumoral radiomics information with DL-derived spatial hierarchical features, transcending the limitations of single-prediction modalities. PCA revealed multidimensional feature contributions: PC1 demonstrated balanced contributions from intratumoral/peritumoral radiomics features and deep learning features, indicating synergistic integration among the three feature classes. PC2 was dominated by DL features, reflecting the significant representational power of 3D CNNs in capturing macroscale tumor spatial heterogeneity (37). The performance of the peritumoral radiomics features was greatest for PC3, which is consistent with the findings from the radiomics analysis in the optimal 6 mm peritumoral region. This synergy underscores the unique value of multidimensional integration in decoding tumor complexity.

In this study, the difference in median survival time between the training and test sets may be partially attributed to the gradual adoption of precise radiotherapy technologies such as image-guided radiotherapy during the multicenter data collection period. Although we controlled confounding factors through uniform inclusion criteria, the retrospective design inherently limits the elimination of selection bias. Notably, prolonged survival itself may reflect therapeutic advancements rather than model prediction bias, providing unique insights into model robustness evaluation within evolving technological contexts. The drop in the combined model’s C-index from the training set (0.77) to the test set (0.73) is a typical manifestation of model generalization across distinct datasets. The training and test sets originate from different medical centers, leading to inevitable discrepancies in patient demographics, treatment details, and data distribution patterns. Despite this decline, the test set C-index of 0.73 still denotes satisfactory predictive performance. To enhance the generalization capability, three key countermeasures were implemented: (1) Integration of L2 regularization (λ=1e-4) with dropout layers (rate=0.4) in the DL framework to prevent overfitting to short-term survival signals; (2) PCA-based feature fusion preserving 95% variance to effectively mitigate multisource data noise; and (3) bootstrap calibration validation demonstrating strong consistency between the predicted probabilities and observed survival rates in the test set. Importantly, the model maintained stable discriminative performance (C-index=0.73) and significant risk stratification capacity (log-rank *P* < 0.001) in the test set, indicating its tolerance to moderate prognostic distribution shifts.

The combined model demonstrated stable risk stratification capabilities across multicenter datasets, enabling individualized prognostic assessments during RT planning for NSCLC patients. Subgroup analyses (by gender, age, histologic subtype, and overall stage) further validated the model’s potential for personalized risk assessment. For example, in the <65 years and ≥65 years subgroups, both the training and test sets exhibited strong risk stratification capacity with consistent HR trends (training set: 7.28 vs. 3.88; test set: 3.37 vs. 5.94). Similarly, in adenocarcinoma and squamous cell carcinoma subgroups, the model maintained stable discriminative power (training set: 4.88 vs. 7.18; test set: 2.94 vs. 5.07), with statistically significant survival differences observed in all subgroups. These findings confirm that despite minor differences between cohorts, the model retains stable prognostic utility, supporting its potential for cross-population clinical application. Risk-based stratification identifies candidates who may benefit from high-dose irradiation, whereas quantitative analysis of peritumoral radiomics features aids in precisely delineating the boundaries of the clinical target volume (CTV).

However, several limitations still exist: (1) retrospective design risks selection bias, necessitating prospective validation; (2) unclear biological basis of the 6-mm extension needs histopathological correlation; (3) due to the lack of detailed records on whether each patient received chemoradiotherapy or radiotherapy alone, we were unable to analyze the potential impact of these two treatment modalities on the study results. Future studies should integrate multi-omics data to unravel imaging–microenvironment molecular correlations, incorporate dosiomics to explore dose–response relationships, and collect detailed records of treatment modalities (e.g., radiotherapy alone vs. chemoradiotherapy) to clarify their impact on outcomes. Additionally, developing explainability tools to demystify model decisions will enhance clinical credibility.

## Conclusion

In conclusion, our multicenter-validated model, which integrates intratumoral, 6-mm peritumoral radiomics, and 3D DL features, significantly improves survival prediction in NSCLC patients receiving radiotherapy. Future efforts will focus on prospective validation and mechanistic exploration of the model’s biological foundations.

## Data Availability

The original contributions presented in the study are included in the article/supplementary material. Further inquiries can be directed to the corresponding authors.
